# Fecal glucocorticoid metabolites as stress biomarkers in common buzzards (*Buteo buteo*) across rehabilitation phases: implications for raptor welfare

**DOI:** 10.3389/fvets.2026.1771891

**Published:** 2026-03-03

**Authors:** Lara-Luisa Grundei, Tanja E. Wolf, Florian Brandes, Karolin Schütte, Fritjof Freise, Ursula Siebert, Chadi Touma, Michael Pees

**Affiliations:** 1 Department of Small Mammal, Reptile and Avian Medicine and Surgery, University of Veterinary Medicine Hannover, Foundation, Hanover, Germany; 2 Department of Behavioral Biology, School of Biology/Chemistry, Osnabrück University, Osnabrück, Germany; 3 Mammal Research Institute, Faculty of Natural and Agricultural Sciences, University of Pretoria, Pretoria, South Africa; 4 Wildlife Rescue and Conservation Center, Sachsenhagen, Germany; 5 Department of Biometry, Epidemiology and Information Processing, University of Veterinary Medicine Hannover, Foundation, Hanover, Germany; 6 Institute of Terrestrial and Aquatic Wildlife Research, University of Veterinary Medicine Hannover, Foundation, Hanover, Germany

**Keywords:** animal welfare, behavior, bird of prey, fGCM, hormone, rehabilitation, stress biomarker, wildlife

## Abstract

Taking wildlife into human care is a balancing act between benefits and harms, as handling and captivity can cause chronic stress that can lead to permanent physiological changes. Therefore, it is necessary to investigate stress levels during the rehabilitation of wild animals such as raptors. Fecal samples from 15 Common Buzzards (*Buteo buteo*) were taken to determine fecal glucocorticoid metabolite concentrations as a biomarker for stress across the rehabilitation phases. Significantly higher concentrations of fecal glucocorticoid metabolites were found during housing phase 1, when the birds were housed in small cages and handled at least once a day for medical treatment, compared to housing phase 2, when they were housed in larger and more undisturbed aviaries. The day of rehabilitation had no significant impact on fecal glucocorticoid metabolite concentrations alone or in interaction with the housing phases. The cause of admission (acute or chronic) and the eventual outcome for the birds (release or euthanasia) also had no statistically significant effect. The results suggest that handling and restraint could be main stressors during rehabilitation and should be critically evaluated throughout the rehabilitation process. Recommendations were derived from our findings to improve the welfare of birds of prey in wildlife rehabilitation.

## 1 Introduction

Taking wildlife into human care is driven by the ethical responsibilities regarding animal welfare and protection, aiming to compensate for the damage caused by human intervention in nature and, ideally, release a healthy animal back into the wild (1–3). However, captivity itself can cause enormous stress for the individual, resulting in a balancing act between benefits and harms, which should be critically evaluated several times during the rehabilitation process for each individual animal (4).

Stress is defined as a threat to the homeostasis of a living being, to which the body responds with an activation of the hypothalamic–pituitary–adrenal (HPA) axis (5). The hypothalamus secretes corticotropin–releasing hormone (CRH), which stimulates the production of adrenocorticotropic hormone (ACTH) in the anterior pituitary gland (6). ACTH then causes the secretion of glucocorticoids in the adrenal gland; the glucocorticoid hormones cortisol and corticosterone have the task to provide glucose and influence the metabolism and the immune system (5, 7, 8). Due to this longer route compared to the direct activation of the adrenal gland via the sympatho-adrenal axis, in which catecholamines are released within seconds, glucocorticoids reach their peak after approximately 30–60 min, depending on the type, duration, and severity of the stressor (5, 9, 10). However, even in stress-free situations, the HPA axis is active at the so-called basal level, with diurnal, seasonal, and highly individual fluctuations (5).

It is known that the stress response of wild animals during captivity is species-specific and that there are few or no data available for many species (11). There have only been a few studies on birds of prey in human care (12–19); however, so far no study has investigated stress and its implications for animal welfare and the outcome of the rehabilitation process in Common Buzzards (*Buteo buteo*) (18). The Common Buzzard is one of the most common and widespread birds of prey in Germany and throughout Europe (20, 21), and is therefore a frequent patient at wildlife rescue centers, making it a suitable and relevant model for research in the field of rehabilitation.

Most rescue centers have strict guidelines to minimize contact with the animals as much as possible to prevent stress or imprinting (22). However, it is necessary to exam, treat, and feed them upon their arrival at the center and at least during the following period until they no longer need to be handled daily. In this phase, birds are typically housed in small individual boxes, often in rooms shared with other birds, and visited frequently by humans (23). The birds are caught at least once a day so that the cage can be cleaned, fresh food and water provided, and they can receive individual medical treatment. Depending on the severity of the cause of their admission they are moved to larger enclosures as soon as possible to minimize contact with humans and give them more space to exercise their flight muscles before being released (24). Limiting their freedom of movement and exposing them to direct, visual, or auditory human contact can contribute to stress and may negatively affect recovery of the wild animals (13, 25–27). Studies on capture and restraint on free-ranging and captive wildlife indicate that stress levels increase as a result of these measures (12, 28–31). However, habituation effects were observed in birds of prey during rehabilitation with increasing time in human care (32). Nevertheless, it remains unclear whether and to what extent the transition from small cages with frequent handling to large aviaries with reduced human contact influences stress hormone levels. Recent studies on owls suggest that the noises in a rescue center are sufficient to cause an increase in stress hormone levels as assessed from fecal samples, underlining the importance of investigating stress and its significance for the rehabilitation process (27).

Observing an animal’s behavior can be highly subjective and may not provide sufficient information about the stress the animal is experiencing (33). To measure objective parameters for stress, such as heart rate or stress hormone concentration, invasive methods have often been used (13, 19, 34). However, using invasive methods in wild animals causes stress itself, as they require handling an untamed animal, and therefore have a direct impact on the data as well as having other limitations, especially in small or endangered species (35, 36). Therefore, detecting glucocorticoid metabolites in feces (fGCM) as a biomarker for stress has become a successful tool in recent years for numerous mammalian and avian species (29, 35, 37–42). Nonetheless, since each species has a very individual excretion and metabolite pattern in addition to the aforementioned time delay in the release of glucocorticoids, a validation must be carried out for each species, either “physiologically” or “biologically,” to find a reliable assay and baseline values for further studies (35, 37). In a previous study we successfully established and validated a method for measuring stress levels using non-invasive markers as a stress indicator in Common Buzzards (43). Using the established Cortisone enzyme immunoassay (EIA), a distinct increase in fGCM concentrations was observed within the first 4 h after the onset of a stress event, reaching peak values between 4 and 8 h, followed by a rapid decline and a return to baseline levels by 16 h after stress onset (43). No sex differences, but diurnal fluctuations in fGCM levels were found (43). An additional degradation experiment established a sample collection interval of 4 h (43). Based on these findings, it is now important to investigate fGCM levels of wild individuals that have been brought into human care due to injury, illness, or weakness, using the established protocol to collect reliable data.

The aim of the present study was therefore to compare fGCM concentrations of wild buzzards temporarily housed in a rescue center during the different phases of rehabilitation to assess the impact of housing conditions on stress levels and to derive recommendations for improving raptor welfare in wildlife rehabilitation. Therefore, we hypothesized that the birds would have a significantly higher stress level in the initial housing phase where the birds were held in small boxes and handled daily due to individual treatment compared to the following housing phase in a more undisturbed aviary. We also assumed that the birds would show decreasing fGCM concentrations due to habituation or recovery and that the cause of admission as well as the outcome (release or euthanasia) may have an impact on the fGCM levels.

## 2 Materials and methods

### 2.1 Animals

Fifteen Common Buzzards (*Buteo buteo*) were admitted to the Wildlife Rescue and Conservation Center (Sachsenhagen, Germany) between November 2023 and March 2025. All 15 buzzards originated from the wild; 12 individuals were admitted directly to the wildlife rescue center, while three were first treated at a specialized Clinic (Department of Small Mammal, Reptile and Avian Medicine and Surgery at the University of Veterinary Medicine Hannover, Hannover, Germany), before being transferred to the center.

Admission reasons included acute trauma (Category A, e.g., collision with vehicles; *n* = 6), and chronic conditions, such as malnutrition or parasitic diseases (Category B, *n* = 9). All animals were at least 6 months old, classified as subadults (*n* = 2) or adults (*n* = 13); the sex was not determined.

Eleven individuals were released after recovery, while four had to be euthanized by administering 300 mg/kg pentobarbital intravenously after anesthesia with isoflurane (induction with 5%, maintenance with 2–3%). All birds were fed daily with day-old chicks, chicken meat (breast or wings), or rats.

### 2.2 Housing phases

**Figure 1:**
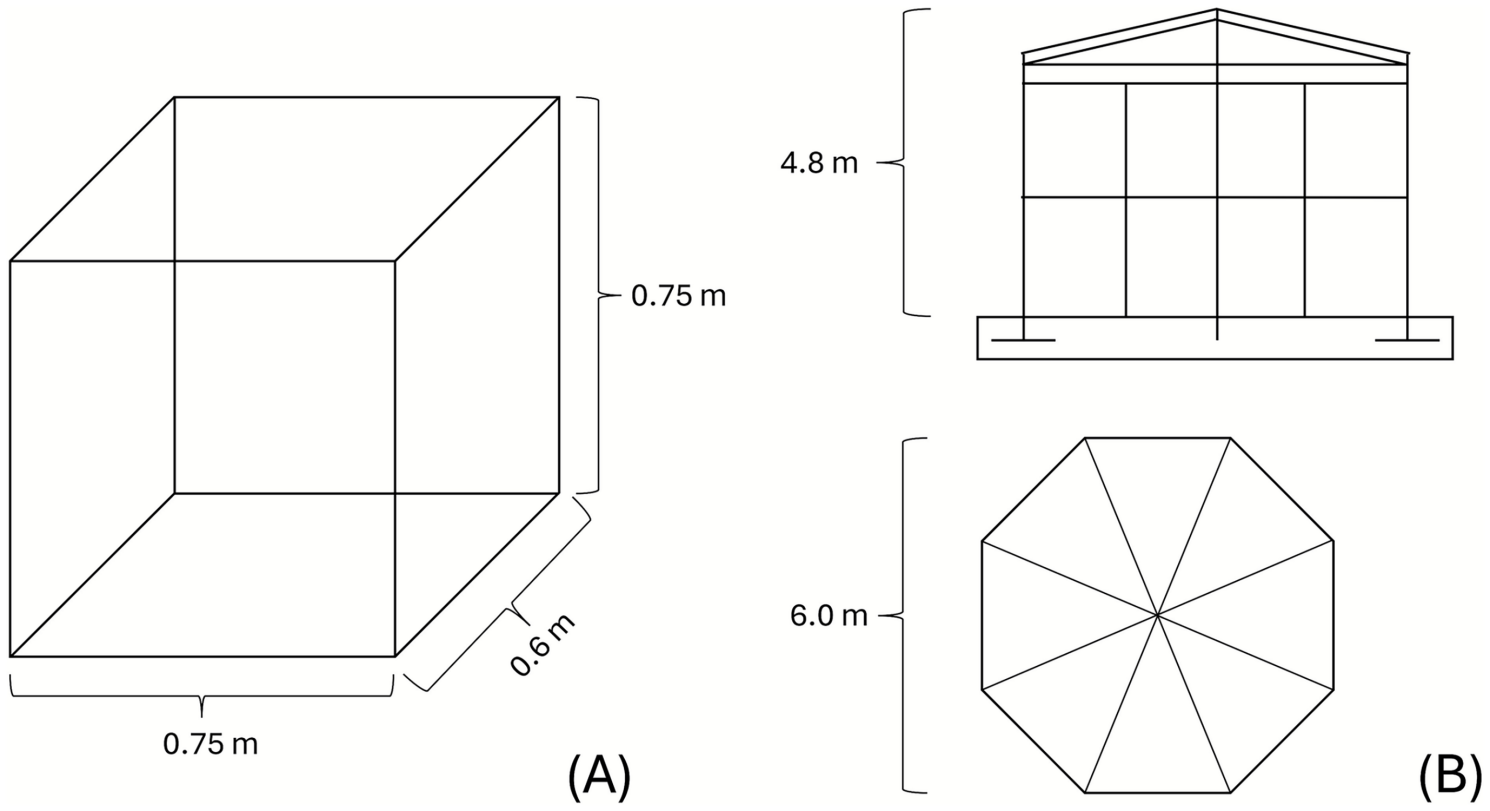
Schematic diagrams of the two housing systems. **(A)** Enclosure in housing phase 1; **(B)** aviary in housing phase 2.

During rehabilitation, birds were successively housed in two different enclosure types:

Housing phase 1 consisted of small cages of 0.45 m^2^ with solid sides and a barred door, lined with paper and equipped with a perch (see Figure 1). These boxes were located either at the rescue center or at the clinic. In this phase, birds received daily medical treatment, including manual handling for medication administration and wound care. This phase lasted at least 5 days for all animals and varied in length depending on the individual treatment (median 11 days, maximum 35 days); for details, see Supplementary materials.

Once daily handling was no longer required, birds were transferred to aviaries at the rescue center for further recovery and stress reduction.

These individual aviaries of approx. 31 m^2^ (housing phase 2) consisted of a wooden enclosure with octagonal layout with closed sides and a mesh roof (height of 4.80 m in the center, see Figure 1). The animals stayed there for at least 10 days until being released.

### 2.3 Sample collection

Upon arrival, the first fecal sample was collected approx. 4 h after the birds had been placed into individual cages for housing phase 1. The box floors were lined with pond liner, and all feces were collected into fecal collection tubes (CVet Kotprobenröhrchen, Covetrus DE GmbH, Düsseldorf, Germany). Subsequently, sampling continued once daily for a minimum of 5 days, always 4 h after the morning cleaning (between 10:00 and 11:00), resulting in sample collection between 14:00 and 15:00.

In housing phase 2, sampling took place every 2 days. A shower tray was placed below the sitting perch, cleaned in the morning, and sampled 4 h later.

All dropped feces were collected with minimal contamination from uric acid and immediately frozen at −18 °C until analysis.

### 2.4 fGCM extraction and analysis

The frozen samples were dried, mortared and fecal glucocorticoid metabolites were extracted mixing 0.05 g fecal powder with 3 mL of 60% methanol according to the protocol described in our previous study (43). For smaller samples between 0.02 and 0.05 g, correspondingly less methanol was added; samples of less than 0.02 g were excluded. Samples were shaken for 30 min on a vibrating plate (Köttermann GmbH, Hänigsen, Germany) and subsequently centrifuged for 20 min at 4500 rpm (Z306, HERMLE Labortechnik GmbH, Wehingen, Germany). The resulting supernatants were transferred to 2 mL tubes (Eppendorf Safe-Lock Tubes, Eppendorf SE, Hamburg, Germany) and refrozen. The extracts were sent to the Laboratory of the Department of Behavioral Biology at Osnabrück University, Germany, for analysis.

Using the validated cortisone enzyme immunoassay, (measuring 4-pregnene-17 *α*, 21-diol-3,11,20-trione-21-HS), fGCM concentrations were detected as described before (42, 43). This assay was developed in-house and performed on microtiter plates according to established protocols (42, 44).

### 2.5 Statistical analysis

Data evaluation was performed with the software Excel for Microsoft 365 MSO (Version 2,506 Build 16.0.18925.20076, Microsoft Corporation, Redmond, WA, United States), SAS Enterprise Guide 7.1 and SAS software 9.4 (SAS Institute Inc., Cary, NC, United States).

Graphs were designed using SAS Enterprise Guide and Excel.

Visual evaluation of the distribution of fGCM (ng/g dry weight) and the residuals of the fitted models using histograms and QQ-plots showed no normal distribution, so the data were log-transformed for the analyses using the natural logarithm.

Descriptive statistics were performed for the original (untransformed) fGCM values.

Different linear mixed models were fitted with restricted maximum likelihood (REML) to the log fGCM data using PROC MIXED to check for the influence of different sources. All models included a random effect for the animal to adjust for individual effects and a statement to model the repeated measurements over the days, where the correlation between two measurements depends on the days between measurements [i.e., some constant to the power of the time difference; in SAS software: SP(POW)]. The influence of the fixed effects (see below) was tested using F-tests. Post-hoc pairwise comparison was performed using Tukey–Kramer adjustment.

To examine changes in the log fGCM concentrations over time, four different models were considered for the main effects: only housing phase, only day of rehabilitation (as a continuous factor), both with and without their interaction. These were used to decide which of the two variables describing the time should be used as a main effect in further modeling.

For the evaluation of associations with admission category (acute vs. chronic) a linear mixed model with fixed effects category, housing phase and their interaction was used. For checking the influence of rehabilitation outcome (release vs. euthanasia) a linear mixed model was fitted with outcome as fixed effect.

Statistical significance was assumed with *p* ≤ 0.05, high statistical significance with *p* ≤ 0.001.

### 2.6 Ethical approval

All samples were obtained non-invasively from wild Common Buzzards (*Buteo buteo*) undergoing routine rehabilitation due to injury or illness. No additional handling, restraint, or prolonged captivity was required for this study. The entire animal study protocol was approved by the Ethics Committee of the Lower Saxony State Office for Consumer Protection and Food Safety (LAVES, trial application no. TV 21A577, 03/18/2021).

## 3 Results

### 3.1 fGCM concentrations over time

**Figure 2:**
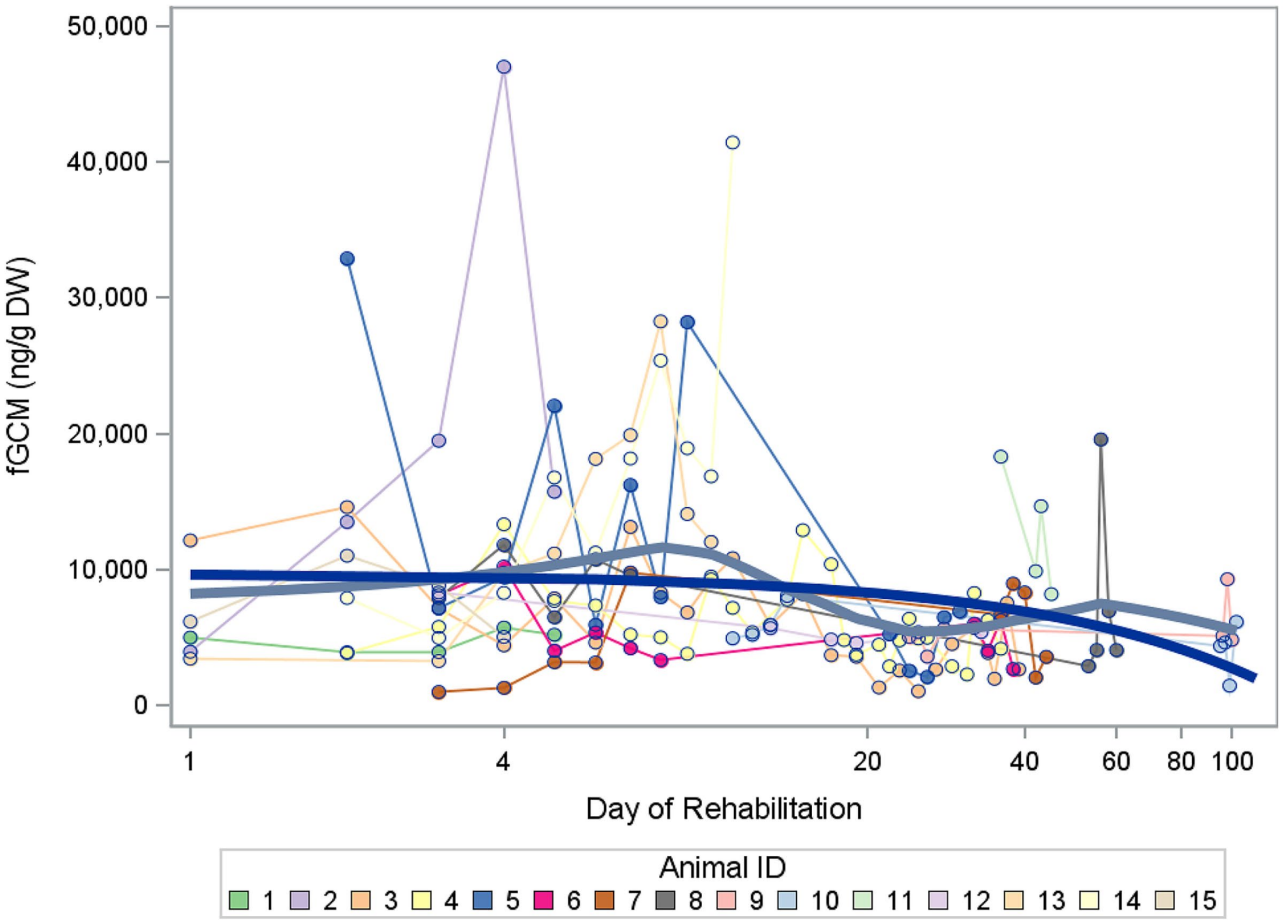
All measured values of fecal glucocorticoid metabolite (fGCM) concentrations in ng/g dry weight (DW) of 15 buzzards over the logarithmic rehabilitation period. The blue line shows the trend over time, the gray line represents the mean line.

As can be seen in Figure 2, there was a wide variation among the buzzards at the start of rehabilitation, with the highest value being 47,015 ng/g fGCM (buzzard 2, day 4) and the lowest value being 1,005 ng/g fGCM (buzzard 7, day 3). The trend line shows a decline in fGCM values the longer the rehabilitation lasts. The total length of rehabilitation varied individually from 5 to 120 days. The latest samples were taken from two animals around day 100 of rehabilitation.

In the mixed model with day of rehabilitation as a fixed effect, this trend in the log fGCM concentrations was not significant (*p* = 0.0663).

### 3.2 fGCM concentrations for different housing phases

**Figure 3:**
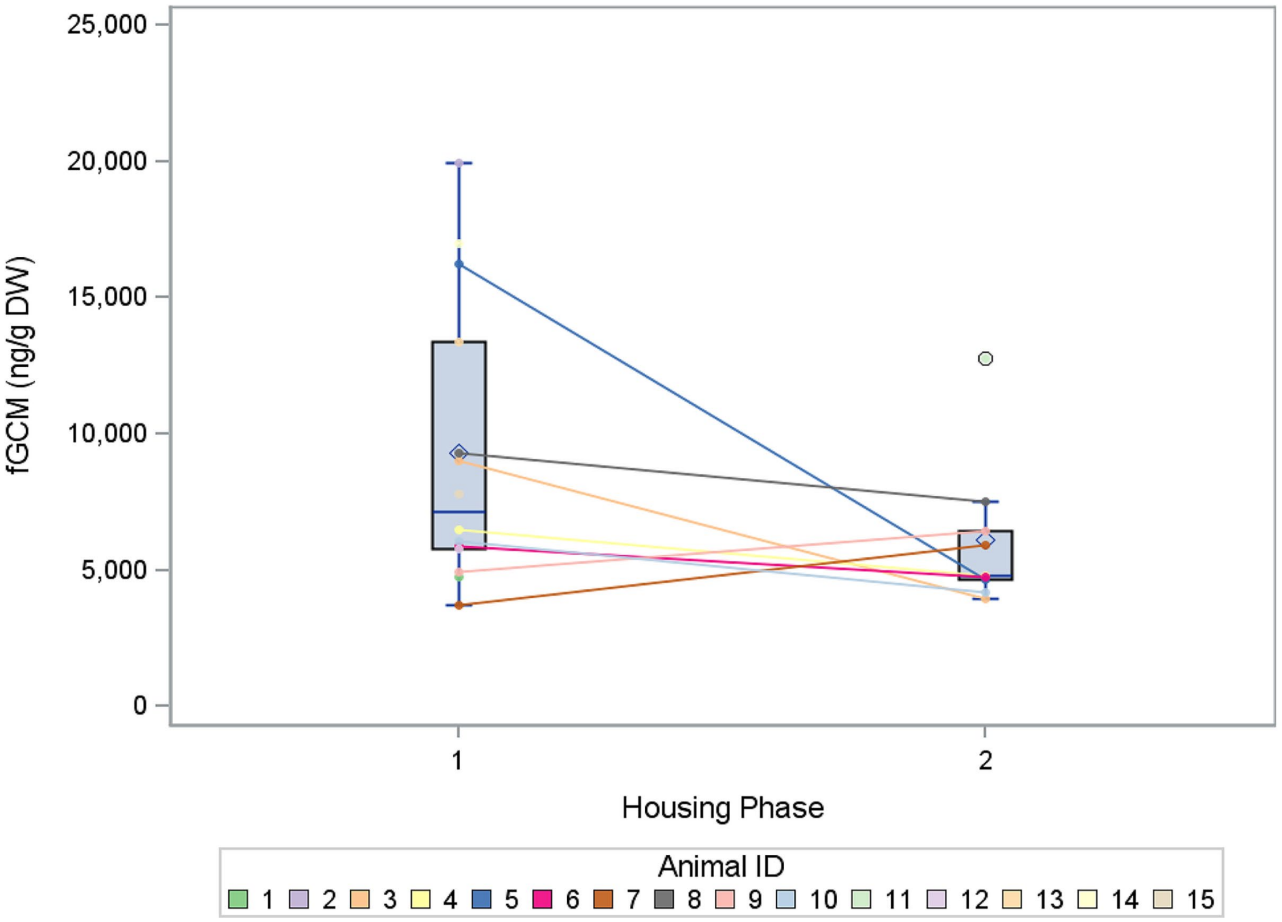
Comparison of the mean concentrations of fecal glucocorticoid metabolites (fGCM) in ng/g dry weight (DW) of 15 buzzards between the two housing phases 1 and 2. The mean values of the buzzards are represented as dots and are connected individually between the two phases.

When comparing the mean fGCM concentrations of buzzards in housing phase 1 and phase 2, the fGCM values decreased by 34% from housing phase 1 (mean = 9,301 ng/g DW) to housing phase 2 (mean = 6,108 ng/g DW). Figure 3 shows the mean values for the individual buzzards when comparing the two housing phases. Significant differences in log fGCM concentrations between housing phases 1 and 2 (*p* = 0.0053) could be found in the mixed model with housing phase only. Estimated mean fGCM concentrations were higher for housing phase 1.

**Figure 4:**
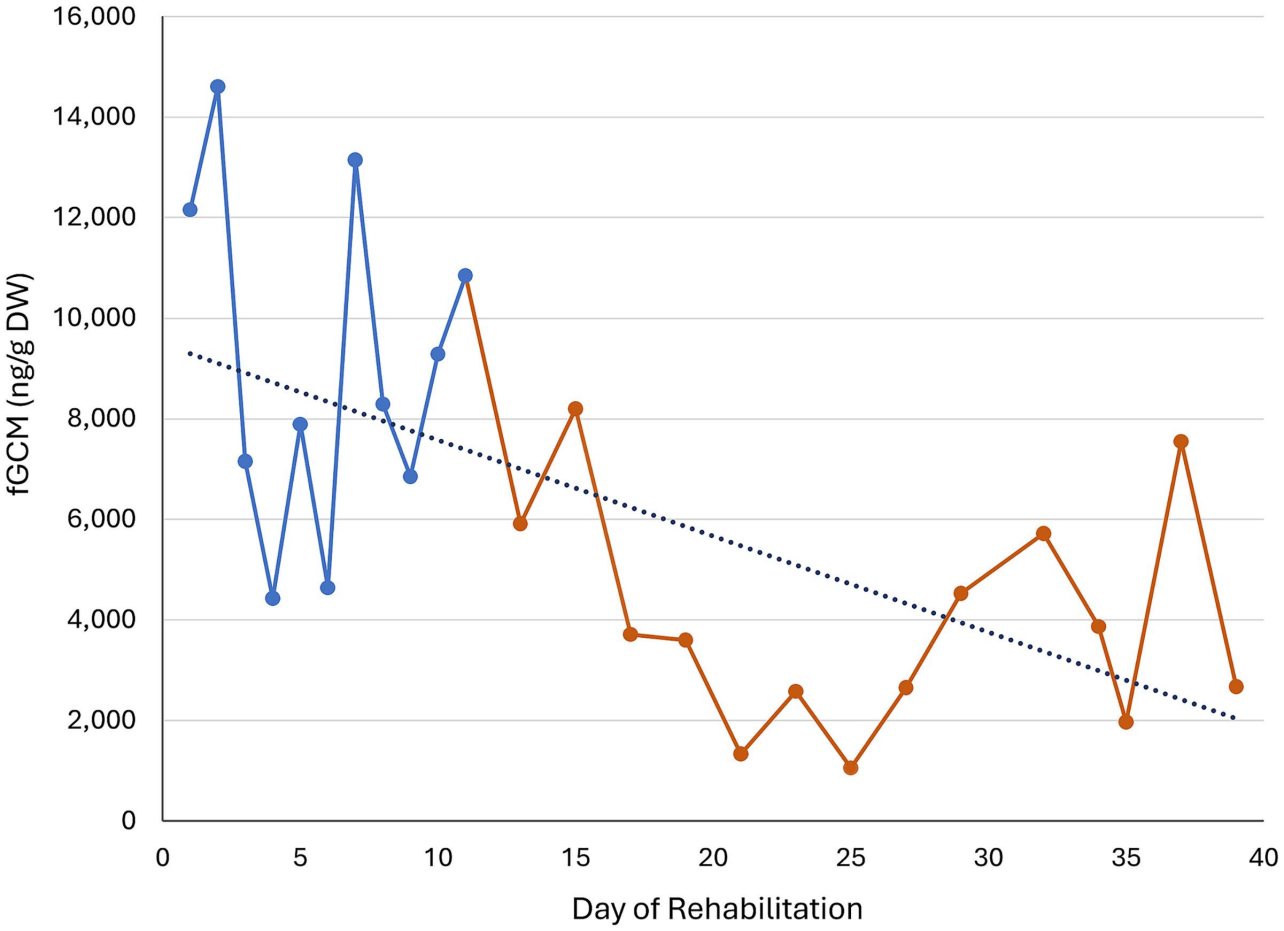
Individual time course of the fecal glucocorticoid metabolite (fGCM) concentrations in ng/g dry weight (DW) of buzzard number 3 during rehabilitation. Housing phase 1 is marked in blue, housing phase 2 in brown. The dotted line shows the trend over time.

While most of the animals showed a decreasing trend (*n* = 13), two animals (buzzard 7 and 9) displayed a slight increase over time. Buzzard number 3 served as an example of a clear decrease in fGCM when switched from housing phase 1 to housing phase 2 (Figure 4). The highest value of buzzard 3 was 14,603 ng/g fGCM at day 2 and the lowest value was 1,056 ng/g at day 25. The bird was admitted due to an acute trauma with broken coracoid bone and released after 114 days of rehabilitation.

### 3.3 Interaction of housing phase and day of rehabilitation

The mixed model with housing phase, day of rehabilitation, and its interaction with the housing phase as fixed effects revealed a significant reduction in log fGCM concentrations from housing phase 1 to housing phase 2 (*p* = 0.0409), but no significant effect of day of rehabilitation (*p* = 0.6491), i.e., no linear trend. The interaction of the two variables was also not statistically significant (*p* = 0.4495). The results of subsections 3.1 to 3.3 led to including the housing phase in the final model in the next section.

### 3.4 fGCM concentrations for different causes of admission

**Figure 5:**
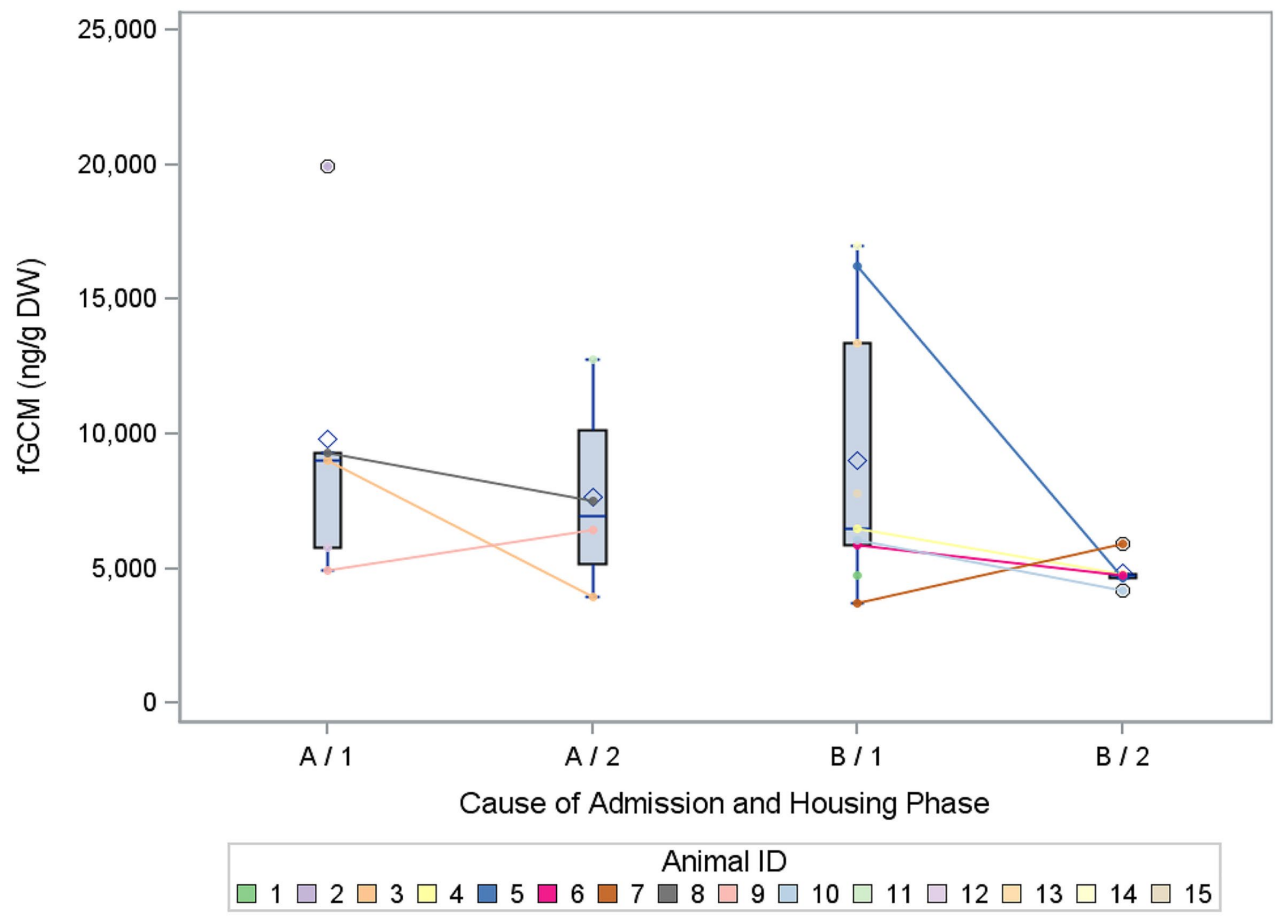
Comparison of the mean concentrations of fecal glucocorticoid metabolites (fGCM) in ng/g dry weight (DW) of 15 buzzards between the categories of cause of admission and the two housing phases. Category A means acute, category B means chronic. The mean values of the buzzards are represented as dots, which are connected individually by a line, when both phases are completed.

A slight deviation in the mean fGCM values between the categories of cause of admission could be seen, with slightly higher values in Category A (acute injury; mean value 9,777 ng/g fGCM) compared to Category B (chronic disease or weakness; mean value 8,466 ng/g fGCM). Figure 5 shows the individual fluctuations in FGCM values between the housing phases. The data showed an overall significant effect of the fixed effects (*p* = 0.0485), but no significant differences in log fGCM concentrations in the causes of admission (*p* = 0.5800) or for the interaction (*p* = 0.9261). Only the housing phase showed a significant effect again (*p* = 0.0058).

### 3.5 fGCM concentrations for different outcomes

**Figure 6:**
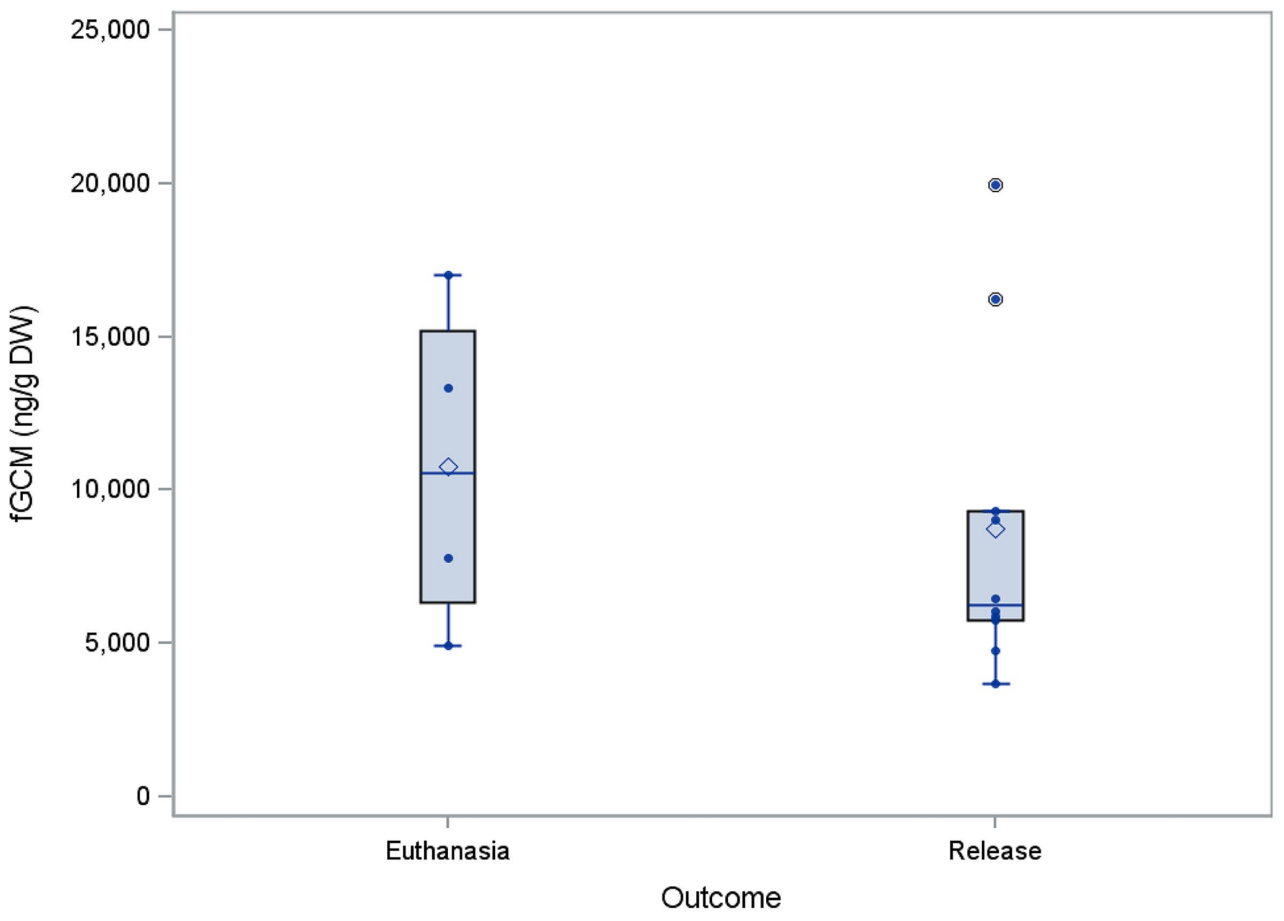
Comparison of the mean concentrations of fecal glucocorticoid metabolites (fGCM) in ng/g dry weight (DW) of 15 buzzards between the outcome. The mean values of the buzzards are represented as dots.

When examining the deviations in fGCM concentrations for different outcomes (release versus euthanasia), we only used log fGCM values from housing phase 1, as most of the euthanized animals did not reach housing phase 2. The four euthanized buzzards showed a higher mean value (10,923 ng/g fGCM) compared to the mean value from the 11 released individuals (8,287 ng/g fGCM), as seen in Figure 6. However, no statistical significance could be determined between the two possible outcomes (*p* = 0.4188).

## 4 Discussion

### 4.1 Fecal glucocorticoid metabolite concentrations across rehabilitation phases

The aim of our study was to detect the impact of housing conditions on stress levels in Common Buzzards during rehabilitation phases and to derive recommendations for birds of prey in temporary human care. The data showed significant overall reduction in fGCM concentrations from the initial phase, where the birds were kept in individual boxes and handled at least once a day, to the following phase, where they were placed in large aviaries without direct human contact.

Both a possible habituation and a recovery effect over the whole rehabilitation period were considered as possible influencing factors. However, day of rehabilitation was not significant when tested in isolation or in the interaction with housing phase in the mixed model.

Instead, the reduction in stress occurred specifically when birds were transferred from box housing to aviaries. This could indicate that the observed decline in fGCM over time was not a habituation or recovery effect, even though time and change in housing systems cannot be definitively distinguished from one another. Nevertheless, there is reason to suspect that human contact and restriction of movement are probably the main stressors during the rehabilitation process of these raptors. Other studies confirm that capture and restraint are extremely stressful for wildlife (12, 28–31) and could even lead to death due to capture myopathy in the worst case (45). D’Amico et al. showed that glucose, total white blood cell count, lymphocytes, and eosinophil levels start to change just 60 min after capture in wild Two-banded Plovers (*Charadrius falklandicus*) (46). Furthermore, Fischer and Romero summarized that capture leads to symptoms of chronic stress, which has negative impact on immune and reproductive systems (11). This could sometimes even lead to permanent physiological alterations (47). In contrast to that, studies on capture stress in wild gray mouse lemurs showed no long-term signs of chronic stress and no significant influence of different handling protocols on the elevation of fGCM levels, but also no habituation to repeated handling (48). However, it can be assumed that human handling, which is necessary for medical treatment during the first rehabilitation period, can have long-term negative effects on the immune system and other physiological processes of the buzzards.

Investigations on animal well-being in zoos revealed that handling armadillos, hedgehogs, and Red-tailed Hawks for education purposes led to increased fGCM values (12). Le Maho emphasized that humoral response to handling stress is not equivalent to absence of stress behavior in geese (33). A study on American Kestrels showed that the mere presence of humans triggered a stress response through an increase in heart rate, plasma corticosterone, and behavioral changes (13). European Starlings experienced a glucocorticoid response in just witnessing a human catching a lure bird, but showed lower corticosterone levels than in witnessing a raptor attack or being caught and handled itself (49). Even human presence in the form of agricultural practice around Barn Owl nesting sites led to an increased basal level of corticosterone and, as a result, to lower body mass of the nestlings (26). Moreover, recent studies suggest that simply playing audio recordings from a rescue center can cause the fGCM levels of Tropical Screech Owls to rise (27). In contrast, tested American Kestrel nestlings did not show any significant change in the HPA axis due to short handling or noise (50). At least in adult animals, in which the HPA axis is fully developed, unlike in young animals, other anthropogenic stressors appear to add to the stress of handling, such as visual and auditory contact with humans.

We did not find evidence of statistically significant habituation in buzzards within the two housing phases, although other studies have reported habituation occurring over longer periods of captivity in some species, e.g., in seven raptor species during rehabilitation, where the corticosterone response to an acute stressor decreased from admission to pre-release samples with increasing length of stay (32). Another study tested whether background radio music in a rehabilitation center could reduce auditory stressors in various wild bird species and found that startle response to extraneous sounds decreased significantly in the observed birds (51). However, a review by Price showed that a bird’s habituation to increased human disturbance is unpredictable because of species and context-specific variations (52). Our results of lacking statistical significance may be due to the fact that the buzzards were not sampled continuously throughout the entire rehabilitation period. However, fecal samples were collected during different time windows within housing phase 2, and no significant linear decrease in fGCM over time was detected. As mentioned above, these results could suggest that the decrease in fGCM concentrations was not caused by habituation or recovery alone but was instead associated with the change in housing conditions, indicating that decreased human contact and a more spacious and more natural environment in housing phase 2 effectively reduced stress levels. However, further studies are needed to reliably distinguish the time factor from the effect.

There were neither significant differences in fGCM concentrations between birds that were later released and those that were euthanized, nor between individuals admitted due to acute injury and those suffering from chronic disease or weakness. This could indicate that acute stress caused by handling and restraint overshadows all other individual conditions. In free-ranging African elephants (*Loxodonta Africana*) acute foot injuries led to a significant increase in fGCM levels, which seemed to be influenced by the duration and severity of the injury (53). Captive Keas (*Nestor notabilis*) also showed significantly higher fGCM levels from the onset of disease symptoms compared to healthy individuals up to 30 days after symptom onset (54). As Baker et al. reviewed, there is some evidence that handling causes stress-induced analgesia in birds, which leads to the assumption that a painful trauma could be muted by the acute handling stress, and this in turn does not increase the fGCM values in comparison to a chronic disease (55). Furthermore, a look at the history of the individual buzzards reveals that animals with acute injuries (*n* = 6) could not always be sampled on the day of admission, but sometimes only days later. In addition, we do not know from the documentation whether the animals were brought to the wildlife rescue center or clinic immediately after the acute trauma happened or whether they were found hours or even days later. This probable time lag between the trauma or onset of illness and admission to the rescue center could have led to a downregulation of the HPA axis due to negative feedback mechanisms (56, 57). Therefore, the categories “acute” versus “chronic” may not be equivalent to acute versus chronic stress, regardless of the fact that a chronic weakness may increase the probability of an acute trauma. The severity of trauma or disease resulted in the euthanasia of four of the 15 buzzards. Three of them were euthanized within housing phase 1 after a maximum of 11 days because of fatal chronic diseases. The fourth was admitted to the clinic with a fractured coracoid and clavicular bone and had to be euthanized at the rescue center after 120 days due to a steady deterioration in its general condition. So not to distort our testing results, the fGCMs were only compared within housing phase 1. In the case of the keas mentioned above, the fGCM concentration also did not differ between sick animals that survived and those that died (54). From a rehabilitation perspective, the absence of significant differences between cause of admission and outcome suggests that all individuals experience similarly high stress during the first days of care, regardless of the underlying state.

**Figure 7:**
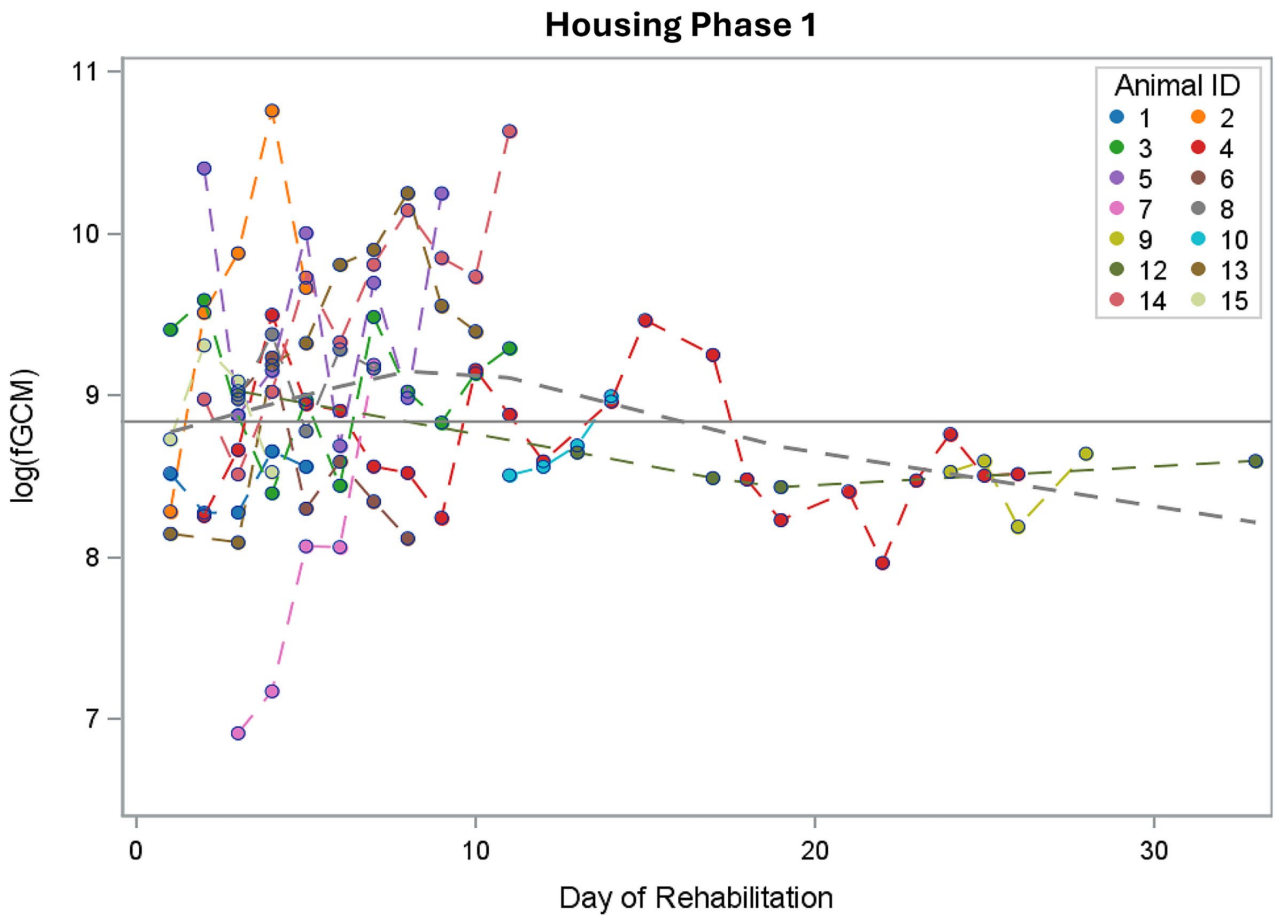
Individual logarithmic fecal glucocorticoid metabolite (log fGCM) concentrations of 15 buzzards during housing phase 1. The gray dashed line represents the mean line; the gray solid line marks the baseline value from the previous study (8.84).

**Figure 8:**
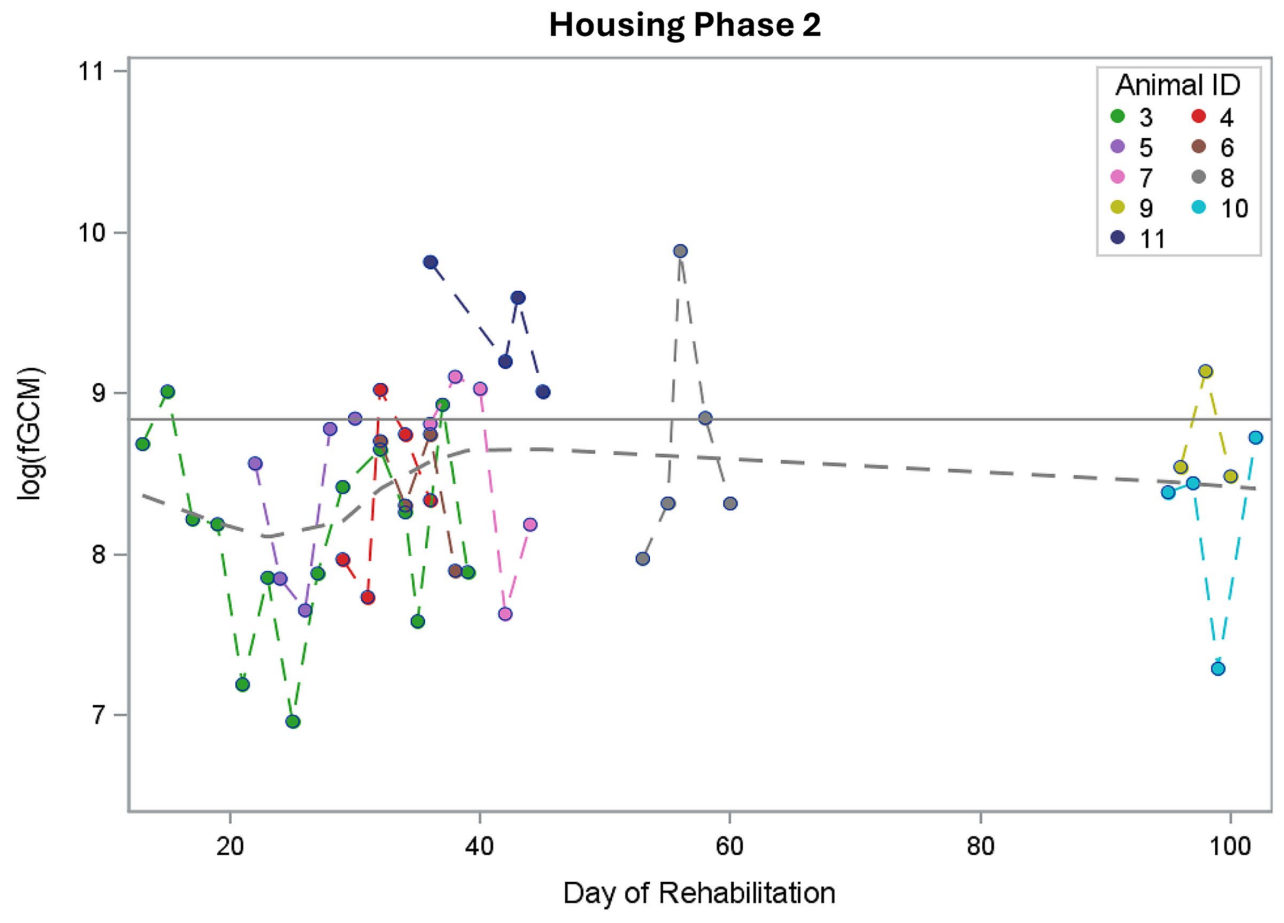
Individual logarithmic fecal glucocorticoid metabolite (log fGCM) concentrations of 15 buzzards during housing phase 2. The gray dashed line represents the mean line; the gray solid line marks the baseline value from the previous study (8.84).

Compared to baseline values for captive buzzards determined in our previous study (6,893 ng/g DW, 8.84 log fGCM) (43), the individual logarithmic fGCM concentrations measured in buzzards admitted to the wildlife rescue center from the wild showed no clear deviation from the baseline values during either housing phase 1 (Figure 7) or housing phase 2 (Figure 8). Overall, fGCM concentrations showed only minor fluctuations around the baseline, with a tendency for the values in housing phase 1 to be slightly above the baseline value and those in housing phase 2 to be rather below it. This may be due to the above-mentioned downregulation of HPA axis. In a study of wild-caught Chukar (*Alectoris chukar*), baseline values and stress-related corticosterone concentrations decreased significantly after 3 to 5 days in captivity, as determined by endogenous negative feedback (58). Another reason could be the four-hour sampling interval in which all droppings were pooled, meaning that different times after handling in the morning were represented in the sample. It can be seen in our previous study that stress decreases rapidly after handling and returns to normal within about 8 h (43). As Legagneux et al. stated, droppings of captured birds do not reflect baseline values of wild free-living birds (59). Even though our first study involved buzzards that were accustomed to humans, wild animals in human care are never completely stress-free because they are not domesticated. Karaer et al. reviewed that fecal glucocorticoids are higher in captive animals than in free-living individuals from the same species (60). For the first study, the animals also had to be transported to an unfamiliar environment and were handled by strangers. To obtain a true baseline for buzzards, samples must be taken non-invasively from wild, undisturbed buzzards.

### 4.2 Implications for raptor welfare in wildlife rehabilitation

Our study is the first to investigate stress during the rehabilitation process in Common Buzzards. This species serves as a role model for raptors in Europe, as animal welfare in raptor rehabilitation has hardly been researched to date. While some studies from North America (18), Canada (2), and Australia (61) have examined the stress physiology of rehabilitated birds of prey, there are currently few comparable data available from European raptor species (62).

The findings suggest that the hands-off policy of most rescue centers is the main factor in reducing stress in birds of prey and that the animals should be placed in quietly situated aviaries as soon as possible. Emphasizing that each handling procedure is extremely stressful, an efficient treatment and cleaning plan should limit the handling frequency to a minimum. For example, the daily medical treatment should be used as an opportunity to clean the cage at the same time. The cage in the initial phase must be of such a size and design that the animal can be caught quickly and safely (63).

The length of rehabilitation in the first phase must be reevaluated at very short intervals to constantly weigh up the high costs against the possible outcome. A poor prognosis or a long healing phase must be weighed against the possibility of *restitutio ad integrum*, the species, age, and life expectancy.

To reduce handling frequency, medication that must be administered several times a day should be carefully weighed in terms of cost and benefit to the animal and, if possible, replaced with medication that needs to be administered less frequently to achieve the same success. Other medical treatments such as wound care, dressing changes, and topical treatments should also be performed as little as possible, but as often as necessary. If possible, oral medication should be administered by adding the medication to the animal’s food rather than by forced oral administration, provided that the animal is eating reliably. To avoid unnecessary handling, only evidence-based medicine and treatments with established efficacy in the affected species should be used. Although these recommendations seem common sense, they should sharpen our focus on individual rehabilitation processes to evaluate and improve them in terms of the potential stress for the animal.

In addition, effective measures to reduce stress should be implemented. Studies have shown a significant reduction in stress-related parameters when using a falcon hood to immobilize various American species of birds of prey (64, 65). Other considerations for reducing stress during human husbandry, such as the use of low-stress medication and enrichment, need to be explored in greater depth.

### 4.3 Limitations

Limiting factors in our results are individual differences and the small sample size. Some samples contained too little material for analysis. This meant that some birds had only three samples per housing phase, while others had up to 30 samples per housing phase. Some birds had to remain in housing phase 1 longer than others, resulting in varying numbers of samples available for analysis.

Another influencing factor may be that the period of sampling in housing phase 2 took place in different periods of time and could not be carried out over the entire period of rehabilitation due to logistical reasons. Therefore, there were large differences in the sampling periods in relation to the duration of rehabilitation.

As the study design followed the normal rehabilitation process of the birds, the effects of the change in housing systems and time cannot be definitely separated from one another. Box housing always took place in the early housing phase, while aviary housing always took place in the late housing phase. To reduce the effect to housing systems only, further studies would have to be conducted in which animals are first housed in aviaries and later in boxes. However, as this would be disadvantageous for birds undergoing rehabilitation, the benefits of the data must be carefully weighed against the welfare of the birds used. Since we only used sick or injured buzzards for the study and cannot compare them with healthy animals, it cannot be ruled out that the elevated fGCM levels are influenced by the animals’ state of health. As mentioned above, studies on healthy wild animals must be critically examined from an ethical perspective.

Although we found no sex differences in our previous study, there could be variations in fGCM regarding sex and season as found in other bird species, which need to be further investigated (15, 36, 66–68). For animal welfare reasons, we refrained from determining the sex in this study, as there is no reliable method for determining the sex of buzzards without requiring direct contact and there is no clear visual sexual dimorphism (69). Furthermore, differences in the age of the animals could also be a factor. Nonetheless, the exact age of the buzzards remained unknown in the case of animals from the wild, although they were classified as subadult or adult, which means no nestlings or fledglings were used for the study.

### 4.4 Outlook

Our findings provide an important foundation for future research on raptor welfare and the management of stress in rehabilitation procedures. The ongoing refinement of measures to enhance animal welfare should remain the foremost objective. Verification of our results with a larger number of test subjects over a longer period of time and other species of birds of prey would be advisable. Further studies with different measures to reduce stress while handling, e.g., background noise, reduced visibility, or chemical restraint, are recommended.

## Data Availability

The original contributions presented in the study are included in the article/Supplementary material, further inquiries can be directed to the corresponding author.
