# Supplementary material for: Fecal glucocorticoid metabolites as stress biomarkers in common buzzards (Buteo buteo) across rehabilitation phases: implications for raptor welfare
Source: Front Vet Sci. 2026 Mar 3;13:1771891. doi: 10.3389/fvets.2026.1771891 (PMC12994232; doi:10.3389/fvets.2026.1771891)
Supplement: Supplementary file 1 [file Data_Sheet_1.docx]

Supplementary Material

# Supplementary Data

Table 1: Results of fecal glucocorticoid metabolite (fGCM) levels during rehabilitation of Common Buzzards (n=15).

| Sample Number | Animal ID | Day of Rehabilitation | fGCM (ng/g DW) | log fGCM | Housing Phase |
| --- | --- | --- | --- | --- | --- |
| 1.1. | 1 | 1 | 5,005 | 8.52 | 1 |
| 1.2. | 1 | 2 | 3,925 | 8.28 | 1 |
| 1.3. | 1 | 3 | 3,930 | 8.28 | 1 |
| 1.4. | 1 | 4 | 5,738 | 8.65 | 1 |
| 1.5. | 1 | 5 | 5,215 | 8.56 | 1 |
| 2.1. | 2 | 1 | 3,956 | 8.28 | 1 |
| 2.2. | 2 | 2 | 13,518 | 9.51 | 1 |
| 2.3. | 2 | 3 | 19,494 | 9.88 | 1 |
| 2.4. | 2 | 4 | 47,015 | 10.76 | 1 |
| 2.5. | 2 | 5 | 15,736 | 9.66 | 1 |
| 3.1. | 3 | 1 | 12,154 | 9.41 | 1 |
| 3.2. | 3 | 2 | 14,604 | 9.59 | 1 |
| 3.3. | 3 | 3 | 7,149 | 8.87 | 1 |
| 3.4. | 3 | 4 | 4,427 | 8.40 | 1 |
| 3.5. | 3 | 5 | 7,889 | 8.97 | 1 |
| 3.6. | 3 | 6 | 4,640 | 8.44 | 1 |
| 3.7. | 3 | 7 | 13,146 | 9.48 | 1 |
| 3.8. | 3 | 8 | 8,291 | 9.02 | 1 |
| 3.9. | 3 | 9 | 6,851 | 8.83 | 1 |
| 3.10. | 3 | 10 | 9,284 | 9.14 | 1 |
| 3.11. | 3 | 11 | 10,845 | 9.29 | 1 |
| 3.12. | 3 | 13 | 5,911 | 8.68 | 2 |
| 3.13. | 3 | 15 | 8,198 | 9.01 | 2 |
| 3.14. | 3 | 17 | 3,708 | 8.22 | 2 |
| 3.15. | 3 | 19 | 3,597 | 8.19 | 2 |
| 3.16. | 3 | 21 | 1,331 | 7.19 | 2 |
| 3.17. | 3 | 23 | 2,578 | 7.85 | 2 |
| 3.18. | 3 | 25 | 1,057 | 6.96 | 2 |
| 3.19. | 3 | 27 | 2,649 | 7.88 | 2 |
| 3.20. | 3 | 29 | 4,527 | 8.42 | 2 |
| 3.21. | 3 | 32 | 5,717 | 8.65 | 2 |
| 3.22. | 3 | 34 | 3,869 | 8.26 | 2 |
| 3.23. | 3 | 35 | 1,968 | 7.59 | 2 |
| 3.24. | 3 | 37 | 7,549 | 8.93 | 2 |
| 3.25. | 3 | 39 | 2,671 | 7.89 | 2 |
| 4.1. | 4 | 2 | 3,854 | 8.26 | 1 |
| 4.2. | 4 | 3 | 5,789 | 8.66 | 1 |
| 4.3. | 4 | 4 | 13,338 | 9.50 | 1 |
| 4.4. | 4 | 5 | 7,693 | 8.95 | 1 |
| 4.5. | 4 | 6 | 7,366 | 8.90 | 1 |
| 4.6. | 4 | 7 | 5,224 | 8.56 | 1 |
| 4.7. | 4 | 8 | 5,021 | 8.52 | 1 |
| 4.8. | 4 | 9 | 3,807 | 8.24 | 1 |
| 4.9. | 4 | 10 | 9,486 | 9.16 | 1 |
| 4.10. | 4 | 11 | 7,190 | 8.88 | 1 |
| 4.11. | 4 | 12 | 5,393 | 8.59 | 1 |
| 4.12. | 4 | 14 | 7,801 | 8.96 | 1 |
| 4.13. | 4 | 15 | 12,905 | 9.47 | 1 |
| 4.14. | 4 | 17 | 10,405 | 9.25 | 1 |
| 4.15. | 4 | 18 | 4,822 | 8.48 | 1 |
| 4.16. | 4 | 19 | 3,750 | 8.23 | 1 |
| 4.17. | 4 | 21 | 4,482 | 8.41 | 1 |
| 4.18. | 4 | 22 | 2,881 | 7.97 | 1 |
| 4.19. | 4 | 23 | 4,794 | 8.48 | 1 |
| 4.20. | 4 | 24 | 6,386 | 8.76 | 1 |
| 4.21. | 4 | 25 | 4,937 | 8.50 | 1 |
| 4.22. | 4 | 26 | 4,989 | 8.51 | 1 |
| 4.23. | 4 | 29 | 2,892 | 7.97 | 2 |
| 4.24. | 4 | 31 | 2,287 | 7.74 | 2 |
| 4.25. | 4 | 32 | 8,279 | 9.02 | 2 |
| 4.26. | 4 | 34 | 6,287 | 8.75 | 2 |
| 4.27. | 4 | 36 | 4,174 | 8.34 | 2 |
| 5.1. | 5 | 2 | 32,916 | 10.40 | 1 |
| 5.2. | 5 | 3 | 7,169 | 8.88 | 1 |
| 5.3. | 5 | 4 | 9,447 | 9.15 | 1 |
| 5.4. | 5 | 5 | 22,071 | 10.00 | 1 |
| 5.5. | 5 | 6 | 5,937 | 8.69 | 1 |
| 5.6. | 5 | 7 | 16,236 | 9.70 | 1 |
| 5.7. | 5 | 8 | 7,975 | 8.98 | 1 |
| 5.8. | 5 | 9 | 28,216 | 10.25 | 1 |
| 5.9. | 5 | 22 | 5,246 | 8.57 | 2 |
| 5.10. | 5 | 24 | 2,565 | 7.85 | 2 |
| 5.11. | 5 | 26 | 2,107 | 7.65 | 2 |
| 5.12. | 5 | 28 | 6,496 | 8.78 | 2 |
| 5.13. | 5 | 30 | 6,928 | 8.84 | 2 |
| 6.1. | 6 | 3 | 8,129 | 9.00 | 1 |
| 6.2. | 6 | 4 | 10,233 | 9.23 | 1 |
| 6.3. | 6 | 5 | 4,023 | 8.30 | 1 |
| 6.4. | 6 | 6 | 5,376 | 8.59 | 1 |
| 6.5. | 6 | 7 | 4,213 | 8.35 | 1 |
| 6.6. | 6 | 8 | 3,354 | 8.12 | 1 |
| 6.7. | 6 | 32 | 6,023 | 8.70 | 2 |
| 6.8. | 6 | 34 | 4,037 | 8.30 | 2 |
| 6.9. | 6 | 36 | 6,286 | 8.75 | 2 |
| 6.10. | 6 | 38 | 2,694 | 7.90 | 2 |
| 7.1. | 7 | 3 | 1,006 | 6.91 | 1 |
| 7.2. | 7 | 4 | 1,303 | 7.17 | 1 |
| 7.3. | 7 | 5 | 3,192 | 8.07 | 1 |
| 7.4. | 7 | 6 | 3,174 | 8.06 | 1 |
| 7.5. | 7 | 7 | 9,787 | 9.19 | 1 |
| 7.6. | 7 | 36 | 6,701 | 8.81 | 2 |
| 7.7. | 7 | 38 | 8,980 | 9.10 | 2 |
| 7.8. | 7 | 40 | 8,336 | 9.03 | 2 |
| 7.9. | 7 | 42 | 2,060 | 7.63 | 2 |
| 7.10. | 7 | 44 | 3,587 | 8.19 | 2 |
| 8.1. | 8 | 3 | 7,921 | 8.98 | 1 |
| 8.2. | 8 | 4 | 11,822 | 9.38 | 1 |
| 8.3. | 8 | 5 | 6,498 | 8.78 | 1 |
| 8.4. | 8 | 6 | 10,763 | 9.28 | 1 |
| 8.5. | 8 | 7 | 9,568 | 9.17 | 1 |
| 8.6. | 8 | 53 | 2,902 | 7.97 | 2 |
| 8.7. | 8 | 55 | 4,091 | 8.32 | 2 |
| 8.8. | 8 | 56 | 19,589 | 9.88 | 2 |
| 8.9. | 8 | 58 | 6,952 | 8.85 | 2 |
| 8.10. | 8 | 60 | 4,086 | 8.32 | 2 |
| 9.1. | 9 | 24 | 5,051 | 8.53 | 1 |
| 9.2. | 9 | 25 | 5,402 | 8.59 | 1 |
| 9.3. | 9 | 26 | 3,604 | 8.19 | 1 |
| 9.4. | 9 | 28 | 5,655 | 8.64 | 1 |
| 9.5. | 9 | 96 | 5,126 | 8.54 | 2 |
| 9.6. | 9 | 98 | 9,296 | 9.14 | 2 |
| 9.7. | 9 | 100 | 4,840 | 8.48 | 2 |
| 10.1. | 10 | 11 | 4,949 | 8.51 | 1 |
| 10.2. | 10 | 12 | 5,211 | 8.56 | 1 |
| 10.3. | 10 | 13 | 5,947 | 8.69 | 1 |
| 10.4. | 10 | 14 | 8,066 | 9.00 | 1 |
| 10.5. | 10 | 95 | 4,384 | 8.39 | 2 |
| 10.6. | 10 | 97 | 4,636 | 8.44 | 2 |
| 10.7. | 10 | 99 | 1,466 | 7.29 | 2 |
| 10.8. | 10 | 102 | 6,158 | 8.73 | 2 |
| 11.1. | 11 | 36 | 18,315 | 9.82 | 2 |
| 11.2. | 11 | 42 | 9,890 | 9.20 | 2 |
| 11.3. | 11 | 43 | 14,672 | 9.59 | 2 |
| 11.4. | 11 | 45 | 8,198 | 9.01 | 2 |
| 12.1. | 12 | 3 | 8,319 | 9.03 | 1 |
| 12.2. | 12 | 13 | 5,694 | 8.65 | 1 |
| 12.3. | 12 | 17 | 4,863 | 8.49 | 1 |
| 12.4. | 12 | 19 | 4,598 | 8.43 | 1 |
| 12.5. | 12 | 33 | 5,407 | 8.60 | 1 |
| 13.1. | 13 | 1 | 3,447 | 8.15 | 1 |
| 13.2. | 13 | 3 | 3,270 | 8.09 | 1 |
| 13.3. | 13 | 4 | 9,770 | 9.19 | 1 |
| 13.4. | 13 | 5 | 11,192 | 9.32 | 1 |
| 13.5. | 13 | 6 | 18,153 | 9.81 | 1 |
| 13.6. | 13 | 7 | 19,908 | 9.90 | 1 |
| 13.7. | 13 | 8 | 28,274 | 10.25 | 1 |
| 13.8. | 13 | 9 | 14,093 | 9.55 | 1 |
| 13.9. | 13 | 10 | 12,040 | 9.40 | 1 |
| 14.1. | 14 | 2 | 7,918 | 8.98 | 1 |
| 14.2. | 14 | 3 | 4,973 | 8.51 | 1 |
| 14.3. | 14 | 4 | 8,287 | 9.02 | 1 |
| 14.4. | 14 | 5 | 16,783 | 9.73 | 1 |
| 14.5. | 14 | 6 | 11,256 | 9.33 | 1 |
| 14.6. | 14 | 7 | 18,180 | 9.81 | 1 |
| 14.7. | 14 | 8 | 25,392 | 10.14 | 1 |
| 14.8. | 14 | 9 | 18,938 | 9.85 | 1 |
| 14.9. | 14 | 10 | 16,867 | 9.73 | 1 |
| 14.10. | 14 | 11 | 41,445 | 10.63 | 1 |
| 15.1. | 15 | 1 | 6,178 | 8.73 | 1 |
| 15.2. | 15 | 2 | 11,030 | 9.31 | 1 |
| 15.3. | 15 | 3 | 8,833 | 9.09 | 1 |
| 15.4. | 15 | 4 | 5,048 | 8.53 | 1 |

Table 2: Results of the initial examination of Buzzards (n=15). The categories for the cause of admission are A (acute) and B (chronic).

| Animal ID | Cause of Admission | | Weight (g) | Age |
| --- | --- | --- | --- | --- |
| 1 | B | Emaciated, lethargic, right foot swollen, feather lice | 710 | adult |
| 2 | A | Lung hemorrhage, blood in the left ear, streaks in the left eye, wheezing | 735 | adult |
| 3 | A | Found unable to fly at the roadside, fractured left coracoid bone | 940 | adult |
| 4 | B | Emaciated, wet, unable to fly, Scaly Face mites, holds left leg up, leg swollen | 700 | adult |
| 5 | B | Severely emaciated, parasitic growths in throat/on tongue | 600 | adult |
| 6 | B | Severely emaciated, unable to fly | 715 | adult |
| 7 | B | Severely emaciated | 520 | subadult |
| 8 | A | Weakened, emaciated, lung hemorrhage, streaks in the right eye, fractured left coracoid bone | 660 | adult |
| 9 | A | Fractured right coracoid bone, old wound and scar on sternum / abdomen | 773 | adult |
| 10 | B | Found lying on the ground in the forest, unable to stand, reduced grasping reflex, severely emaciated | 524 | adult |
| 11 | A | Collision with car, bleeding in both eyes, standing unsteadily, lung hemorrhage | 985 | adult |
| 12 | A | Collision with car, hematoma on chest, right wing hanging down, fractured right radius | 658 | adult |
| 13 | B | Found lying on the ground, unable to stand properly, right foot swollen, old wound on forehead, emaciated | 595 | adult |
| 14 | B | Severely emaciated, streaks in both eyes, lungs and abdominal air sac opaque on X-ray | 570 | subadult |
| 15 | B | Unable to fly, enlargements on the left elbow and left side of the chest, abdomen protruding and lumpy, enlarged liver, suspected tuberculosis | 630 | adult |

Table 3: Rehabilitation process of Buzzards (n=15). In the outcome, R stands for release and E for euthanasia.

| Animal ID | Days in Housing Phase 1 | Total Days in Rehabilitation | Outcome |
| --- | --- | --- | --- |
| 1 | 5 | 74 | R |
| 2 | 5 | 35 | R |
| 3 | 11 | 114 | R |
| 4 | 26 | 105 | R |
| 5 | 9 | 54 | R |
| 6 | 9 | 83 | R |
| 7 | 10 | 78 | R |
| 8 | 20 | 94 | R |
| 9 | 30 | 120 | E |
| 10 | 16 | 108 | R |
| 11 | unknown | 89 | R |
| 12 | 35 | 42 | R |
| 13 | 11 | 11 | E |
| 14 | 12 | 12 | E |
| 15 | 5 | 5 | E |

# Supplementary Figures

#
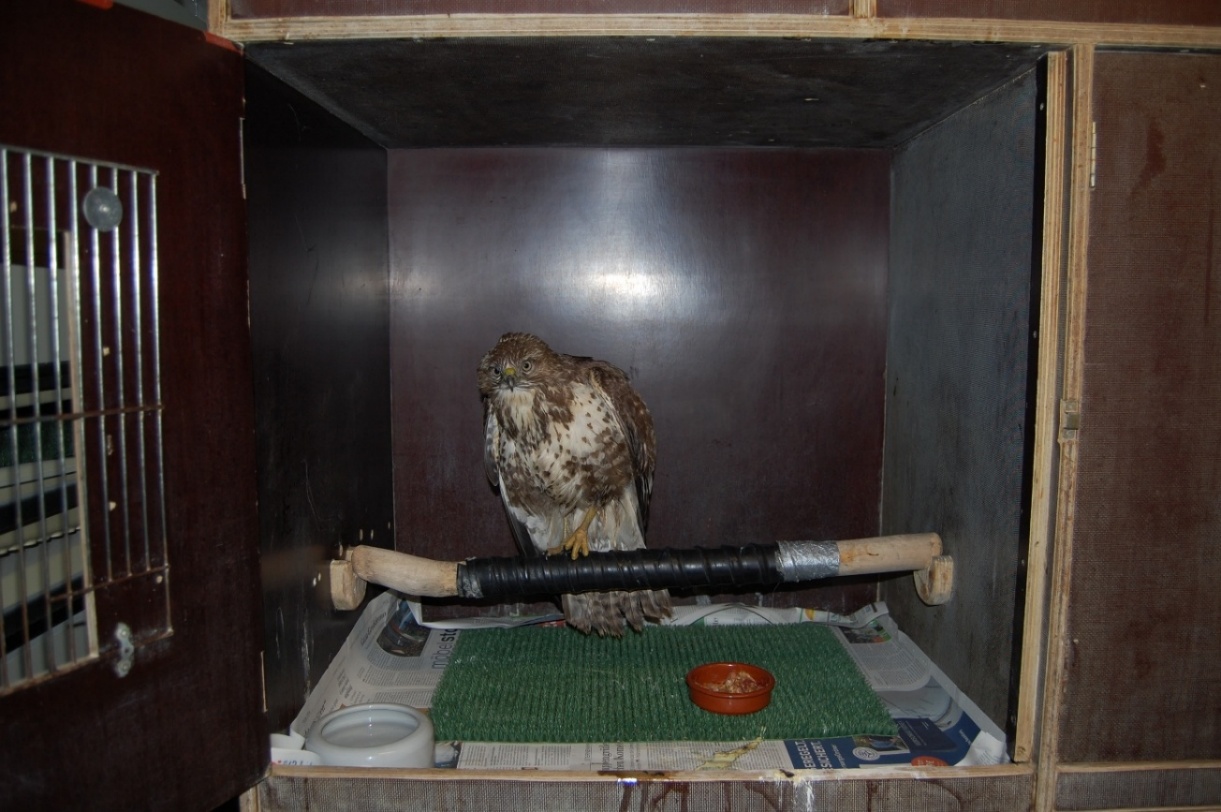


Supplementary Figure 1: Image of the enclosure in housing phase 1.


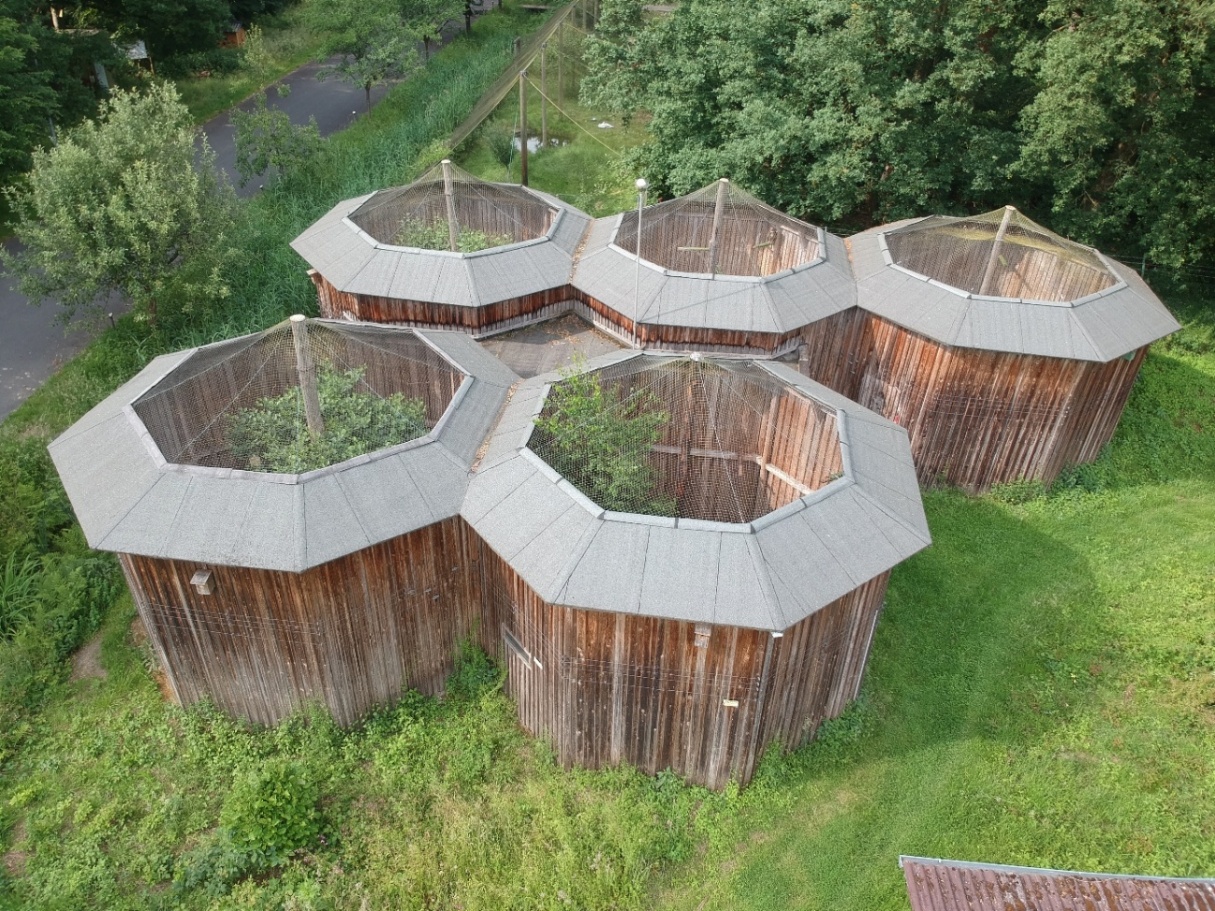


Supplementary Figure 2: Image of the aviaries in housing phase 2.
